# Improving Visible Light Positioning Accuracy Using Particle Swarm Optimization (PSO) for Deep Learning Hyperparameter Updating in Received Signal Strength (RSS)-Based Convolutional Neural Network (CNN)

**DOI:** 10.3390/s25237256

**Published:** 2025-11-28

**Authors:** Chun-Ming Chang, Yuan-Zeng Lin, Chi-Wai Chow

**Affiliations:** Department of Photonics & Graduate Institute of Electro-Optical Engineering, College of Electrical and Computer Engineering, National Yang Ming Chiao Tung University, Hsinchu 30010, Taiwan

**Keywords:** visible light positioning (VLP), convolutional neural network (CNN), received signal strength (RSS), particle swarm optimization (PSO)

## Abstract

Visible light positioning (VLP) has emerged as a promising indoor positioning technology, owing to its high accuracy and cost-effectiveness. In practical scenarios, signal attenuation, multiple light reflections, or light-deficient regions, particularly near room corners or furniture, can significantly degrade the light quality. In addition, the non-uniform light distribution by light-emitting diode (LED) luminaires can also introduce errors in VLP estimation. To mitigate these challenges, recent studies have increasingly explored the use of machine learning (ML) techniques to model the complex nonlinear characteristics of indoor optical channels and improve VLP performance. Convolutional neural networks (CNNs) have demonstrated strong potential in reducing positioning errors and improving system robustness under non-ideal lighting conditions. However, the performance of CNN-based systems is highly sensitive to their hyperparameters, including learning rate, dropout rate, batch size, and optimizer selection. Manual tuning of these parameters is not only time-consuming but also often suboptimal, particularly when models are applied to new or dynamic environments. Therefore, there is a growing need for automated optimization techniques that can adaptively determine optimal model configurations for VLP tasks. In this work, we propose and demonstrate a VLP system that integrates received signal strength (RSS) signal pre-processing, a CNN, and particle swarm optimization (PSO) for automated hyperparameter tuning. In the proof-of-concept VLP experiment, three different height layer planes (i.e., 200, 225, and 250 cm) are employed for the comparison of three different ML models, including linear regression (LR), an artificial neural network (ANN), and a CNN. For instance, the mean positioning error of a CNN + pre-processing model at the 200 cm receiver (Rx)-plane reduces from 9.83 cm to 5.72 cm. This represents an improvement of 41.81%. By employing a CNN + pre-processing + PSO, the mean error can be further reduced to 4.93 cm. These findings demonstrate that integrating PSO-based hyperparameter tuning with a CNN and RSS pre-processing significantly enhances positioning accuracy, reliability, and model robustness. This approach offers a scalable and effective solution for real-world indoor positioning applications in smart buildings and Internet of Things (IoT) environments.

## 1. Introduction

Over the past decades, optical wireless communication (OWC) and visible light communication (VLC) have attracted increasing attention as promising wireless technologies. OWC and VLC leverage the wide and unregulated optical spectrum; hence, they can address the issue of spectral congestion in conventional radio frequency (RF) communication systems [1,2,3,4,5,6,7,8,9,10,11,12,13,14,15,16]. In addition, VLC signals do not interfere with RF signals, making VLC an effective complement to the existing RF communication systems. Moreover, the integration of VLC with the existing light-emitting diode (LED) lighting infrastructure further enables simultaneous illumination and data transmission, offering the advantages of reducing the deployment cost and power consumption [13,17,18,19,20,21,22,23,24]. Given the wide deployment of these LEDs in indoor environments, such as offices and factories, dense VLC attocell networks can be realized by these LEDs to achieve negligible electromagnetic interference (EMI). Moreover, since VLC signals cannot penetrate walls and provide highly directional communication links, VLC inherently offers enhanced physical layer security to privacy. Beyond the high-data-rate transmission, VLC systems can also support high-accuracy indoor visible light positioning (VLP) [25,26,27,28].

VLP is regarded as a promising technique for indoor positioning and localization, offering many advantages such as high spatial accuracy, immunity to EMI, and low deployment cost. With the rapid growth of smart buildings and Internet of Things (IoT) applications, the demand for reliable indoor positioning systems has become more and more critical. As discussed above, VLP leverages the widespread deployment of LED lighting infrastructure, enabling positioning functionality through various measurement schemes, including proximity [29], time-of-arrival (TOA) [30], angle-of-arrival (AOA) [31,32], time-difference-of-arrival (TDOA) [33], and received signal strength (RSS) [34,35].

Among these schemes, RSS-based positioning has garnered considerable attention owing to its simplicity, compatibility with low-cost photodiodes, and ease of integration with existing LED lighting infrastructure. Despite these advantages, RSS-based VLP systems face several practical challenges. In real-world practical scenarios, signal attenuation, multiple light reflections, or light-deficient regions, particularly near room corners or furniture, can significantly degrade the light signal quality. In addition, the non-uniform light distribution by LED luminaires can also introduce errors in VLP estimation. To mitigate these challenges, recent studies have increasingly explored the use of machine learning (ML) techniques to model the complex and nonlinear relationship between RSS data and physical coordinates. These models employed include linear regression (LR) [36,37], kernel ridge regression [38], pre-processing-based approaches [39,40], artificial neural networks (ANN) [41,42], long short-term memory neural network (LSTM-NN) [43,44,45], and convolutional neural network (CNN) [46].

CNNs have shown strong potential in learning spatial RSS patterns and improving positioning accuracy. For example, previous work [46] has shown that combining a CNN with signal normalization and RSS enhancement can reduce positioning errors and improve system robustness under non-ideal lighting conditions. In addition, polynomial feature modeling has been proposed to capture nonlinear dependencies in signal-based localization. Polynomial regression effectively represents higher-order and interaction relationships that linear models cannot capture. Such nonlinear modeling concepts provide the theoretical foundation for our feature-expansion strategy in RSS-based VLP [47].

However, the performance of CNN-based systems is highly sensitive to their hyperparameters, including learning rate, dropout rate, batch size, and optimizer selection. Manual tuning of these parameters is not only time-consuming but also often suboptimal, particularly when models are applied to new or dynamic environments. Therefore, there is a growing need for automated optimization techniques that can adaptively determine optimal model configurations for VLP tasks. Recent studies have continued to advance VLP technology by improving RSS-based ML models and analyzing the impact of LED calibration on localization accuracy. Several works published between 2023 and 2025 have also explored different optimization strategies, such as particle swarm optimization (PSO). This illustrates a growing trend of using automated hyperparameter tuning to achieve more reliable and robust indoor positioning systems [48,49,50]. In recent years, various hyperparameter optimization algorithms, such as grid search, genetic algorithms (GA), and Bayesian optimization, have been applied to deep learning models. These approaches, however, present different trade-offs between search efficiency, computational cost, and convergence stability. Grid search exhaustively explores all parameter combinations and is time-consuming. GA and Bayesian optimization provide more flexible search mechanisms but often require complex parameter tuning and sequential evaluations, which limit their scalability in real-time or large-scale applications [51,52]. The PSO algorithm offers a lightweight, gradient-free, and parallelizable solution that can achieve near-optimal convergence with fewer function evaluations. These characteristics make PSO particularly suitable for VLP systems, where model optimization needs to balance training efficiency and localization accuracy.

In this work, we propose and demonstrate a VLP system that integrates RSS signal pre-processing, a CNN, and PSO for automated hyperparameter tuning. The optimization process in PSO is guided by minimizing the root mean square error (RMSE) between the predicted and actual receiver (Rx) coordinates. RMSE is a widely adopted performance metric in regression-based localization because it directly reflects the Euclidean distance error and penalizes large deviations more strongly than mean absolute error (MAE). This metric has been extensively applied in VLP studies to quantify positioning accuracy and optimize model parameters [53]. PSO is a population-based stochastic optimization algorithm inspired by the social behavior of birds [54,55]. It has been widely applied in ML and signal processing due to its simplicity and fast convergence. In the proof-of-concept VLP experiment, three different-height Rx planes (i.e., 200, 225, and 250 cm) are employed for the comparison of three different ML models (i.e., LR, ANN, CNN). For instance, the mean positioning error of a CNN + pre-processing model at the 200 cm Rx-plane reduces from 9.83 cm to 5.72 cm, representing an improvement of 41.81%. By employing a CNN + pre-processing + PSO, the mean error can be further reduced to 4.93 cm. These findings demonstrate that integrating PSO-based hyperparameter tuning with CNN and RSS pre-processing significantly enhances positioning accuracy, reliability, and model robustness. This approach offers a scalable and effective solution for real-world indoor positioning applications in smart buildings and IoT environments. The innovation of this work is the combination of the CNN algorithm with the design of the system and the combination of techniques, e.g., pre-processing, PSO, which allow it to be less sensitive to hyperparameters and to automatically optimize these parameters. In addition, while advanced architectures such as ResNet or RetinaNet could potentially improve positioning performance, our study intentionally employs a lightweight CNN with a computationally efficient framework suitable for low-latency indoor VLP applications.

## 2. Algorithms of RSS Data Pre-Processing, CNN Model, PSO, and Proof-of-Concept Experiment

The proposed and demonstrated VLP system architecture here is composed of three core components: (i) RSS-based signal pre-processing, (ii) a CNN model for positioning, and (iii) a PSO algorithm for hyperparameter tuning. This section provides a detailed explanation of each component and its role in improving positioning accuracy, particularly in light-deficient indoor environments.

### 2.1. RSS Data Acquisition and Pre-Processing

**Figure 1:**
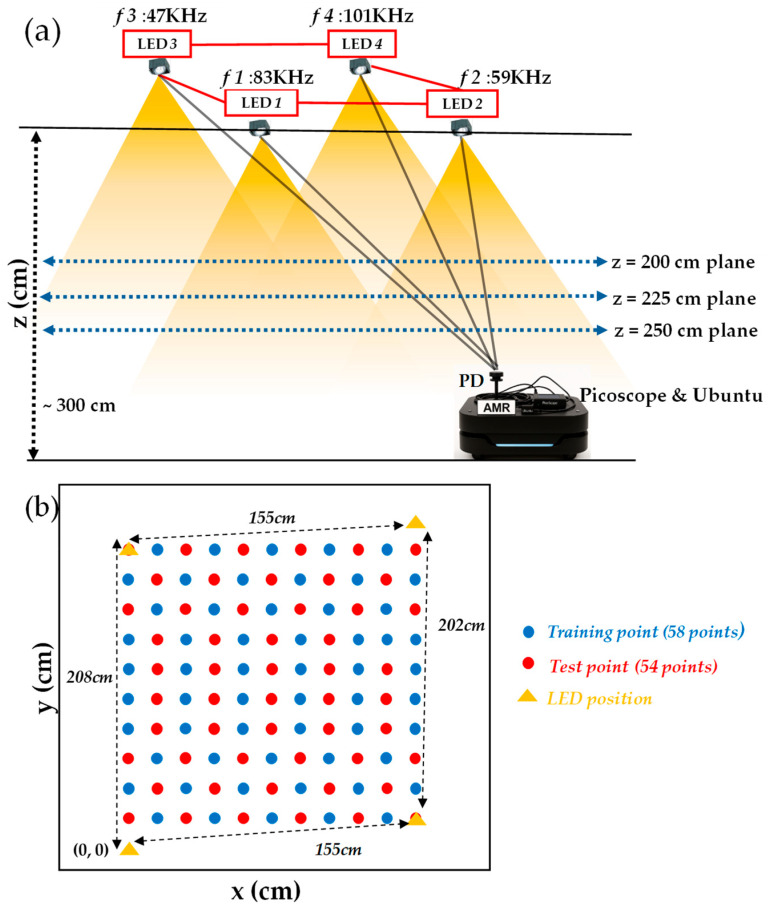
(**a**) Experimental configuration of the proposed RSS-based VLP system. The receiver (Rx) planes are positioned at heights of 200 cm, 225 cm, and 250 cm below the LED panel. LED: light-emitting diode; PD: photodiode. (**b**) VLP Rx plane layout showing locations of training points, test points, and LEDs. The coordinate origin (0,0) is located at the bottom-left corner, where the *x*-axis increases toward the right, and the *y*-axis increases upward. It is worth noting that due to the constraints of the room, the four LEDs are not arranged in a perfect square.

Figure 1a shows the proof-of-concept experimental configuration of the proposed RSS-based VLP. It comprises four LED lamps (TOA LDL030C, 30 W) and each modulated at distinct Manchester-coded carrier frequencies of 47 kHz, 59 kHz, 83 kHz, and 101 kHz, respectively. Each ceiling-mounted LED lamp provides wide-angle illumination used in indoor lighting. The lamps operate with a broad beam divergence (approximately 110–120°) suitable for uniform coverage of the Rx plane. Each LED is 30 W. Here, each location yields 14 input features derived from the RSS values of four LEDs and their polynomial feature expansion, as illustrated in Equation (2). The use of odd frequencies allows VLP signal transmission with negligible interference by harmonics. The received RSS data are collected using an Autonomous Mobile Robot (AMR) equipped with a photodiode (PD, Thorlabs^®^ PDA100A2) with a wavelength detection range from 320 to 1100 nm and an embedded transimpedance amplifier. The analog output is sampled using a real-time oscilloscope (Pico Technology PicoScope^®^ 5432D) and processed on a laptop computer running the Ubuntu operating system. The PD is on different Rx planes at heights of 200 cm, 225 cm, and 250 cm, respectively. Figure 1b shows the Rx plane with the locations of training points, test points, and LEDs. It is worth noting that due to the constraints of the room, the four LEDs are not arranged in a perfect square. The spacing between different LEDs is also shown in Figure 1b.

To improve model performance and reduce environmental inconsistencies, pre-processing is applied. First, Z-score normalization is performed per frequency channel to standardize the input distribution, as shown in Equation (1),

(1) $$z=\frac{\left(p_{i}-\mu_{i}\right)}{\sigma_{i}}$$

where *p_i_* denotes the RSS value of the *i*-th LED at a specific position, while $\mu_{i}$, *σ_i_* represent the mean and standard deviation of the RSS values for that LED across the training dataset. This normalization step mitigates the bias introduced by uneven LED mounting heights and ensures consistent input scaling for deep learning models, which improves training stability and convergence.

In addition to normalization, the input features are explicitly expanded to capture both linear and nonlinear relationships from RSS measurements. Each sample consists of 14 input features used by both the ANN and CNN models. These features include first-order terms, interaction (cross) terms, and second-order (quadratic) terms, as shown in Equation (2).

(2) $$v=\left[p_{1},p_{2},p_{3},p_{4},p_{1}^{2},p_{1}p_{2},p_{1}p_{3},p_{1}p_{4},p_{2}^{2},p_{2}p_{3},p_{2}p_{4},p_{3}^{2},p_{3}p_{4},p_{4}^{2}\right]$$

Polynomial feature expansion is an important approach for capturing nonlinear relationships by augmenting first-order inputs with interaction and higher-order terms. Polynomial regression can effectively model nonlinear dependencies that linear models cannot represent [47]. In this work, we explicitly include first-order, cross, and quadratic terms (i.e., Equation (2)) to better approximate the nonlinear mapping from RSS to spatial coordinates. Hence, the positioning accuracy can be improved.

This combination enables the models to capture direct, cross, and higher-order signal dependencies, thereby enhancing localization performance from raw RSS data. Beyond normalization and feature expansion, geometric correction is applied to address signal degradation caused by vertical distance differences. Since the PD is placed at different height planes, including 200 cm, 225 cm, and 250 cm, away from the LED plane, an RSS enhancement algorithm [46] is adapted to compensate for geometric attenuation. Specifically, the method incorporates the field-of-view (FOV) angle and vertical distance between the transmitter (Tx) and receiver (Rx), as shown in Equation (3),

(3) $${RSS}_{Enhanced}=RSS+\frac{\left(h_{std}-h_{tar}\right)\tan\theta }{h_{std}\tan\theta }\alpha$$

where $h_{std}$ and $h_{tar}$ represent the standard and target plane heights, respectively. *θ* denotes the FOV half-angle of the photodiode, and *α* is a scaling coefficient determined empirically. By adjusting the raw RSS values according to height-based geometric loss, this enhancement technique helps to unify signal characteristics across planes and strengthens the CNN’s ability to learn spatially consistent features. These combined enhancements ensure that the input data for the ML models is both normalized and geometrically aligned, forming a robust foundation for accurate position estimation.

### 2.2. Model Design and Training

Here, we evaluate three models for VLP: LR, ANNs, and CNNs for comparison. The LR used is similar to in [37]. The ANN architecture consists of an input layer followed by three hidden layers, each containing 64, 32, or 16 neurons, depending on the hyperparameter settings, and a final output layer. The hidden layers use the Rectified Linear Unit (ReLU) activation function to introduce nonlinearity and enhance learning capability. To prevent overfitting, a dropout mechanism with a dropout rate of 0.1 is applied after each hidden layer, which helps the model generalize better to unseen data. The ANN model is trained using the Adam optimizer with a batch size of 32, as it provides efficient convergence and stable gradient updates for RSS-based regression tasks. This configuration is selected after preliminary experiments to balance among the model complexity, convergence speed, and generalization ability. The fully connected feed-forward structure of the ANN enables the model to approximate complex nonlinear mappings between RSS inputs and location coordinates.

**Figure 2:**
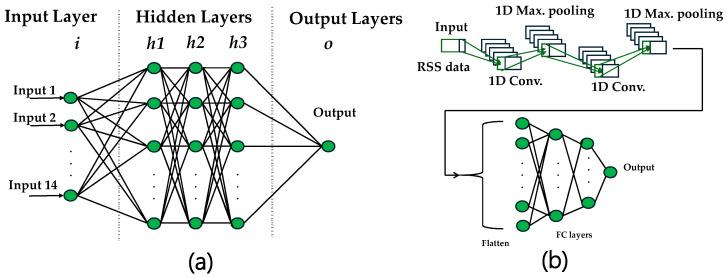
Neural network architectures: (**a**) ANN model; (**b**) CNN model.

The CNN model is designed to capture positioning patterns and spatial relationships in the RSS inputs. It begins with a one-dimensional convolutional layer (kernel size = 3, number of filters = 8 or 32), followed by max-pooling layers to reduce dimensionality and retain the most significant features. These features are then flattened and passed through two dense layers and a final output layer that predicts the (x, y) coordinates. To prevent overfitting, dropout layers with a dropout rate of 0.1 are applied after each dense layer. The CNN model is trained using the Adam optimizer with a batch size of 32, which provides efficient and stable convergence during parameter optimization for spatial RSS features. The local receptive field of the convolutional layer allows the CNN to exploit neighboring relationships in RSS data, improving robustness in noisy or spatially heterogeneous signal conditions. Figure 2 illustrates the architecture of both the ANN and CNN models. The ANN model adopts a fully connected feed-forward structure, while the CNN model features convolutional and pooling layers tailored for spatial RSS vector processing.

Compared with ANN and LR, the CNN model demonstrates superior performance in environments with irregular signal propagation, multipath effects, or light-deficient regions due to its ability to extract localized spatial features. However, since deep learning models are sensitive to hyperparameter settings, we incorporated PSO to automatically optimize key parameters, including learning rate, batch size, and dropout ratio, thereby enhancing positioning accuracy and robustness across different spatial planes. The PSO algorithm is used to minimize the RMSE between the predicted and ground-truth coordinates. RMSE is a widely adopted performance metric in regression-based positioning systems because it directly reflects the Euclidean distance error and penalizes large deviations more strongly than MAE. This property makes RMSE particularly suitable for VLP, where large outlier errors can significantly degrade localization stability and accuracy [53]. Consequently, RMSE serves as both the evaluation criterion and the objective function for PSO-based hyperparameter optimization.

### 2.3. Hyperparameter Optimization via PSO

Hyperparameter updating plays an important role in enhancing the performance of deep learning models. Key parameters, including the learning rate, batch size, dropout rate, and optimizer type (e.g., Adam or SGD), significantly affect the model convergence behavior, predictive accuracy, and ability to generalize across diverse datasets. Traditional methods, such as grid search or manual trial-and-error, are often time-consuming and computationally expensive, particularly when dealing with deep architectures or high-dimensional hyperparameter spaces. To overcome these challenges, we adopt the PSO algorithm, which has demonstrated strong effectiveness in solving nonlinear, multidimensional optimization problems without relying on gradient-based information, which makes it particularly well-suited for tuning deep learning hyperparameters.

In the PSO algorithm, each solution candidate—referred to as a particle—represents a unique combination of hyperparameters. These particles iteratively explore the solution space by adjusting their velocity and position based on both their individual historical best (personal best) and the best performance observed across the swarm (global best). The PSO iteratively updates each particle’s position and velocity based on both individual experience and the swarm’s collective knowledge, as described by Equations (4) and (5),

(4) $$v_{i}^{t+1}=\omega v_{i}^{t}+c_{1}r_{1}\left(p_{i}^{best}-x_{i}^{t}\right)+c_{2}r_{2}\left(g^{best}-x_{i}^{t}\right)$$

(5) $$x_{i}^{t+1}=x_{i}^{t}+{v_{i}}^{t+1}$$

where $v_{i}^{t+1}$: velocity of particle *i* at iteration *t*,

$x_{i}^{t}$: current position of particle *i* at iteration *t*,

ω: inertia weighting,

$p_{i}^{best}$: personal best of particle *i*,

$g^{best}$: global best of the group,

$\left(c_{1},c_{2}\right)$: acceleration coefficients,

$\left(r_{1},r_{2}\right)$: uniformly distributed random number between 0 and 1.

These equations guide each particle toward regions of the search space with potentially better solutions while balancing exploration and exploitation. Through iterative updates, the swarm collectively converges toward optimal or near-optimal hyperparameter configurations that maximize model performance.

**Figure 3:**
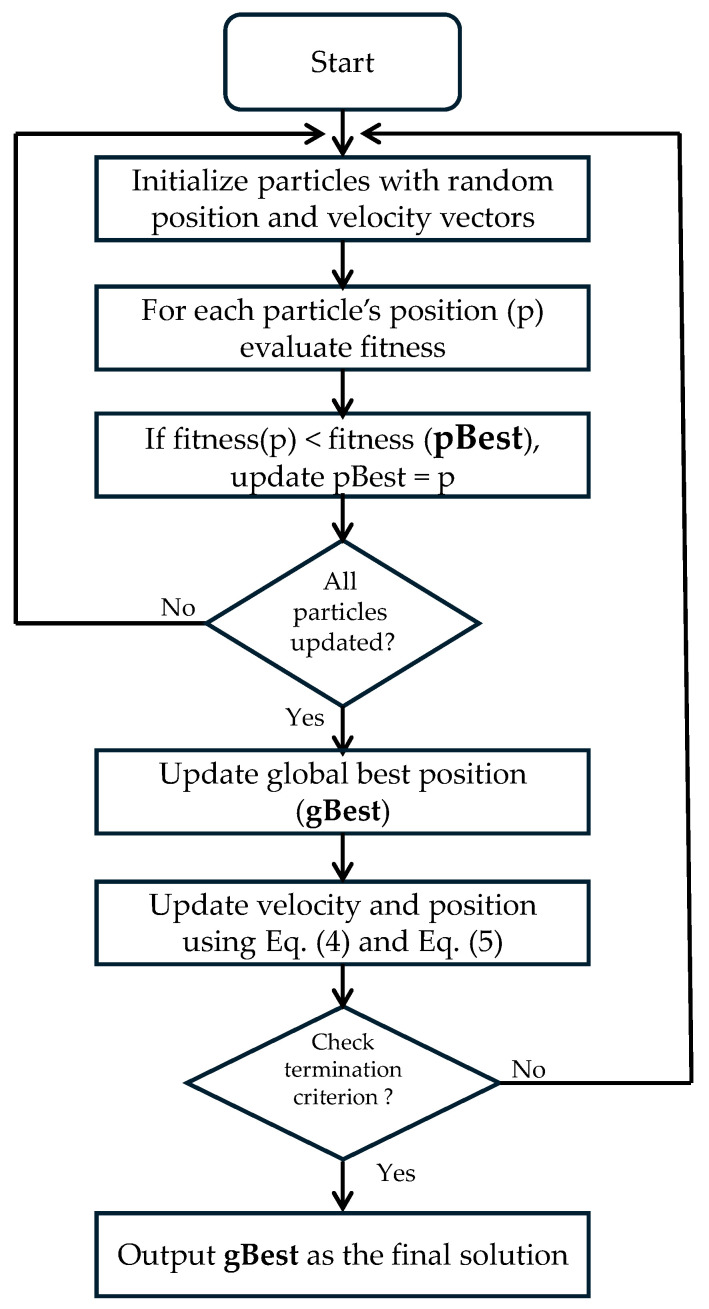
Flow chart depicting the PSO algorithm.

As shown in Figure 3, PSO starts by randomly initializing particles. Each evaluates fitness, updates its personal best (pBest), and the global best (gBest) is determined. The particles’ positions and velocities are then updated iteratively via Equations (4) and (5) until convergence, yielding gBest as the optimal result.

In our implementation, the fitness function is defined as the RMSE between the predicted and actual (x, y) coordinates. PSO searches for hyperparameter combinations that minimize this error across validation data. To ensure convergence and avoid overfitting, we include early stopping criteria during model training and constrain each hyperparameter within predefined bounds. The PSO is configured with 30 particles over 50 iterations, and standard swarm parameters are used: inertia weight (w) = 0.7, cognitive coefficient (c_1_) = 1.5, and social coefficient (c_2_) = 1.5. These parameter settings are widely adopted in PSO studies for achieving a good balance between exploration and exploitation and ensuring stable convergence [51,52]. The RMSE is adopted as the fitness function to guide the search for optimal parameter combinations. Each candidate set of hyperparameters is evaluated using the ANN and CNN frameworks, and the best-performing set is selected. Four key hyperparameters are selected for optimization in Table 1. By integrating PSO into our training pipeline, we eliminate the need for manual hyperparameter selection and achieve more consistent performance across different environmental settings. The dropout rate and learning rate are randomly initialized within the specified continuous ranges, while the batch size and optimizer are selected from discrete candidate sets. The resulting models demonstrate improved positioning accuracy and robustness, especially in light-deficient regions or varying receiver planes. PSO not only reduces manual effort in hyperparameter selection but also significantly enhances the model reliability and cross-plane consistency. A static learning rate is used within each model training process during PSO. In addition, PSO particles are initialized using constrained random uniform sampling within predefined ranges for learning rate, dropout rate, batch size, and optimizer type.

All model training and PSO experiments are conducted on a laptop computer equipped with an AMD Ryzen 7 2600 CPU, NVIDIA RTX 5060 GPU, AMD Radeon 780 M GPU, and 32 GB RAM, running Windows 11 and CUDA Toolkit 11.2. The PSO configuration used 30 particles and 50 iterations, and achieved a 15–20% reduction in the final RMSE. This demonstrates that the PSO-based optimization provides a good trade-off between computational cost and accuracy improvement in the proposed CNN-based VLP system.

It is also worth noting that for each receiver plane, the PSO with 30 particles and 50 iterations performed approximately 1500 automated hyperparameter evaluations per model. In contrast, conducting an equivalent number of manual tuning experiments would require days of continuous human effort. By automating this process, PSO effectively transfers human labor into computational optimization, finishing the full multi-plane optimization within five days. Moreover, each PSO iteration employs randomized yet constrained parameter initialization for batch size, learning rate, dropout rate, and optimizer type, ensuring broad search-space coverage and robust convergence.

## 3. Results and Discussion

### 3.1. Evaluation of LR, ANN, and CNN Models with and Without Pre-Processing

We compare and evaluate our RSS-based VLP results using three models—LR, an ANN, and a CNN. To enhance robustness and mitigate issues such as signal variation and light-deficient regions, we applied data pre-processing techniques to normalize the input RSS features prior to model training. Table 2 presents the positioning errors (mean ± standard deviation) for all models, both with and without pre-processing, across three planes: 200 cm, 225 cm, and 250 cm. Among these models, the CNN model outperforms them across all planes. The CNN model without pre-processing yields the mean positioning errors of 9.83 cm, 8.99 cm, and 5.97 cm at the 200 cm, 225 cm, and 250 cm planes, respectively. After applying the pre-processing, these mean errors are significantly reduced to 5.72 cm, 5.46 cm, and 4.85 cm, respectively.

The effect of data pre-processing is proven clearly. When pre-processing is applied, the performance of all models improves significantly. For instance, the mean positioning error of the CNN + pre-processing model at the 200 cm Rx-plane reduces from 9.83 cm to 5.72 cm. This represents an improvement of 41.81% when calculated using Equation (6). The CNN + pre-processing model exhibits improvements of 41.81%, 39.27%, and 18.76% at 200 cm, 225 cm, and 250 cm Rx-planes, respectively. Similarly, the mean positioning error of the ANN + pre-processing model reduces from 9.86 cm to 6.36 cm at the 200 cm Rx-plane, and from 7.36 cm to 5.75 cm at the 225 cm Rx-plane. LR also benefits from pre-processing, and the mean positioning error reduces from 11.33 cm to 8.79 cm at the 200 cm Rx-plane, and from 8.52 cm to 8.10 cm at the 225 cm Rx-plane. Table 2 summarizes the mean and standard deviation of the positioning errors for all models with and without pre-processing.

(6) $$\mathrm{Improvement}\;(\%)=\frac{\;E_{model}-\left(\;E_{\left\{mode+Pre.\right\}}\right)}{\;E_{model}}\times 100\%$$

Equation (6) calculates the percentage improvement using only the mean values in Table 2. In Equation (6), *E_model_* denotes the mean position error before pre-processing using the LR, ANN, or CNN models, and *E_{mode+Pre.}_* denotes the mean positioning error after pre-processing with the corresponding models (i.e., LR + Pre., ANN + Pre., or CNN + Pre.).

### 3.2. Performance Enhancement Through PSO-Based Hyperparameter Tuning

To further improve model accuracy and robustness, PSO is employed to automatically tune key hyperparameters for both the ANN and CNN models. These hyperparameters include learning rate, dropout rate, batch size, and the optimizer type, and the number of convolutional filters (for CNN), which can significantly affect model convergence and generalization. All evaluations are conducted using the same pre-processed RSS datasets to ensure consistency, reliability, and fair comparison among different models. As shown in Table 3, the CNN + pre-processing + PSO model achieves notable improvements in mean positioning errors: 4.93 cm, 4.53 cm, and 3.87 cm for the 200 cm, 225 cm, and 250 cm Rx-planes, respectively. Compared with the CNN + pre-processing without PSO, these results show the accuracy gains of 13.81%, 17.03%, and 20.21% at the 200 cm, 225 cm, and 250 cm Rx-planes, respectively. These improvements confirm that PSO enables the CNN model to better exploit the spatial features embedded in RSS data, compared to conventional manual tuning methods. The reduction in error also indicates that PSO contributes to better generalization performance by selecting more effective hyperparameter combinations. Similarly, the ANN model combined with pre-processing and PSO (ANN + Pre. + PSO) also yields consistent improvements. The mean positioning errors decrease to 6.05 cm, 4.62 cm, and 4.09 cm at the 200 cm, 225 cm, and 250 cm Rx-planes, corresponding to relative accuracy gains of 4.87%, 19.65%, and 23.84%, respectively. The accuracy gain is calculated using Equation (7). These findings suggest that the impact of PSO is particularly pronounced for ANN models, potentially due to their higher sensitivity to initial hyperparameter configurations and greater risk of overfitting. The consistent performance gains across all spatial planes highlight the robustness of the PSO-enhanced models. Unlike manual tuning or grid search, which may fail to capture optimal hyperparameter interactions, the swarm-based search in PSO allows for dynamic exploration and exploitation of the search space, adjusting parameters based on both local and global knowledge. This results in models that not only converge faster but also maintain high accuracy under diverse environmental conditions. Table 3 summarizes the mean and standard deviation of the positioning errors of the ANN and CNN Models using pre-processing with and without PSO.

(7) $$\mathrm{Accuracy}\;\mathrm{Gain}\;(\%)=\frac{\;E_{\left\{model+Pre.\right\}}-\left(\;E_{\left\{mode+Pre.+PSO\right\}}\right)}{\;E_{\left\{model+Pre.\right\}}}\times 100\%$$

Equation (7) calculates the accuracy gain (%) using only the mean values in Table 3. In Equation (7), *E*_{*mode*__+*Pre.*}_ denotes the mean position error after pre-processing using different models (i.e., LR + Pre., ANN + Pre., or CNN + Pre.), and *E*_{*mode*__+*Pre.*+*PSO*}_ denotes the corresponding mean positioning error after applying pre-processing and PSO.

In conclusion, the integration of PSO with deep learning architectures significantly boosts positioning performance in RSS-based VLP systems. It enhances both the accuracy and stability of ANN and CNN models by intelligently tuning critical parameters. These improvements are particularly valuable in practical VLP deployments, where environmental variations and signal irregularities present ongoing challenges. PSO thus serves as an effective tool to maximize the capability of deep learning models in real-world indoor localization scenarios, particularly in scenarios characterized by challenging signal propagation and dynamic environmental variability.

### 3.3. Robustness Analysis Across Spatial Planes and Model Architectures

**Figure 4:**
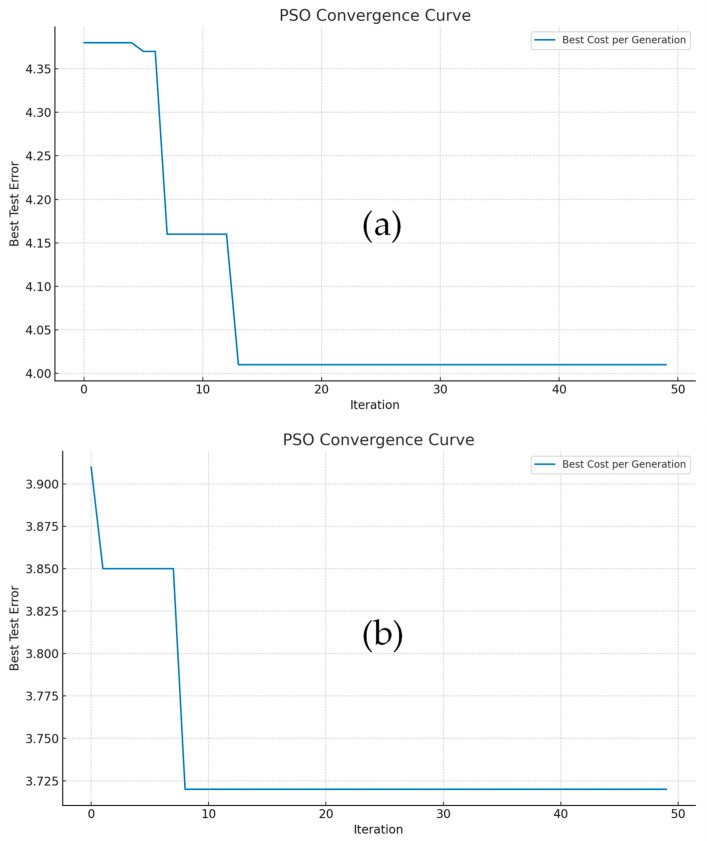
PSO convergence curves for the (**a**) ANN and (**b**) CNN models at the 250 cm height plane. The best test error, computed as the minimum test positioning error across all PSO model evaluations according to Equation (8).

The consistent improvements observed across all Rx-planes indicate that CNN models tuned with PSO are not only more accurate but also demonstrate enhanced robustness against signal variations induced by distance and environmental noise. Moreover, the benefits of PSO are more pronounced in deeper models (e.g., CNN), suggesting a higher sensitivity of convolutional architectures to hyperparameter choices. These findings suggest that PSO is particularly beneficial for deeper architectures and can serve as an effective hyperparameter optimization strategy for VLP tasks. To visually evaluate the effectiveness of PSO, we present the convergence curves for ANN and CNN models trained with 30 particles over 50 iterations. Figure 4a,b show PSO convergence curves against iterations for the ANN and CNN models at 250 cm Rx-plane. They show the progressive reduction in the best test error across generations, highlighting the effective exploration–exploitation balance achieved by the particle swarm. For both ANN and CNN configurations, the PSO algorithm rapidly reduces the test error within 15 iterations and then stabilizes, indicating successful hyperparameter search and reduced risk of overfitting. Additionally, the CNN model exhibits a faster and more substantial error reduction in early iterations compared to the ANN model, highlighting the increased optimization sensitivity and greater potential for improvement in deeper neural architectures, such as CNN. Because PSO evaluates K different hyperparameter configurations, each having a corresponding test error. To quantify the best achievable performance during PSO, the best test error used in Figure 4 is defined as the minimum test positioning error obtained among all PSO evaluations, expressed using Equation (8),

(8) $$E_{best}=\underset{k=1,2,\ldots n}{\min}E_{test}^{k}$$

where K: the total number of PSO evaluations (1500 for 30 × 50 iterations),

$E_{\mathrm{test}}^{k}$: denotes the test positioning error of the k-th model evaluation.

### 3.4. CDF-Based Performance Comparison

**Figure 5:**
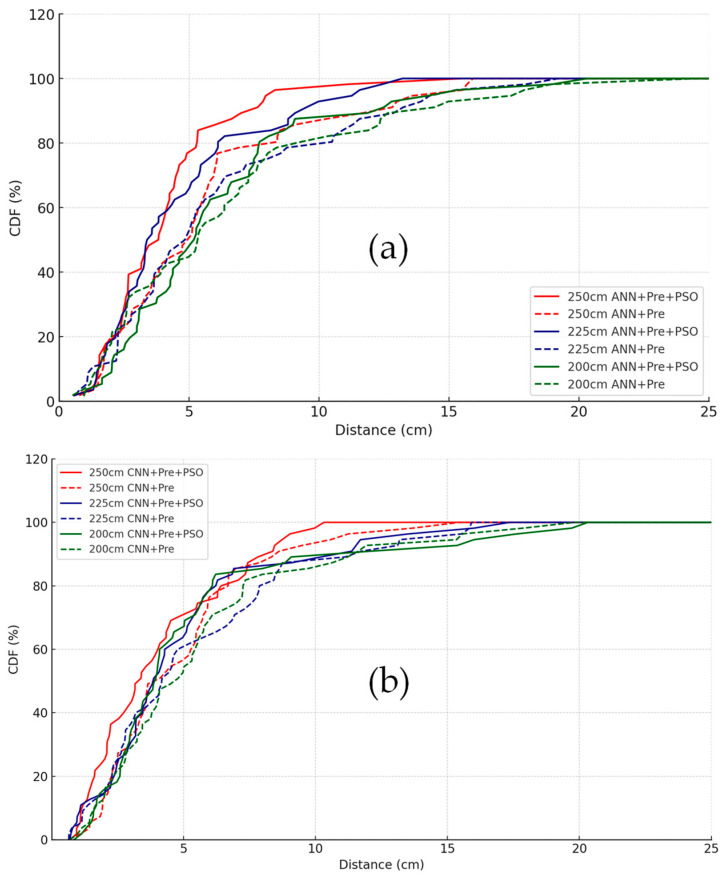
CDF of positioning errors using the (**a**) ANN and (**b**) CNN models with and without PSO-based tuning under three Rx-planes (200 cm, 225 cm, 250 cm). The CDF values are obtained according to Equation (11).

To further validate the effectiveness of PSO-based optimization, Figure 5a,b present the cumulative distribution function (CDF) curves of positioning errors under different configurations and measurement heights (200 cm, 225 cm, and 250 cm) for the ANN and CNN models, respectively. The PSO-optimized models exhibit steeper CDF curves, indicating improved robustness and more tightly clustered positioning errors. For the ANN model shown in Figure 5a, at the 200 cm Rx-plane, the 90% positioning error is reduced from 12.25 cm (ANN + Pre-processing) to 7.32 cm (ANN + Pre-processing + PSO), representing a 40% reduction. Similarly, the CNN model shown in Figure 5b demonstrates significant performance gains with PSO tuning. At 250 cm, the CNN + Pre-processing + PSO configuration achieves 90% cumulative accuracy within 6.52 cm, compared to 9.74 cm for the non-optimized version, illustrating the advantage of PSO in achieving more consistent and reliable positioning performance across all tested Rx-planes. To obtain the CDF curves shown in Figure 5, the two-dimensional Euclidean positioning error is first calculated for each test sample. For the n-th test point, the individual positioning error $e_{n}$ is defined using Equation (9), and the average positioning error across all test samples is computed using Equation (10). This average error definition is used throughout Table 2 and Table 3 and in the Conclusions. The empirical cumulative distribution function (CDF) at a distance threshold d is obtained using Equation (11).

(9) $$e_{n}=\sqrt{{\left(x_{n}^{\left\{pred\right\}}-x_{n}^{\left\{true\right\}}\right)}^{2}+{\left(y_{n}^{\left\{pred\right\}}-y_{n}^{\left\{true\right\}}\right)}^{2}\;}$$

(10) $$\bar{e}=\frac{1}{N}\sum_{n=1}^{N}e_{n}$$

(11) $$F\left(d\right)=\frac{1}{N}\sum_{n=1}^{N}I\left(e_{n}\leq d\right)$$

where *N*: the total number of test samples,

I(·): denotes the indicator function,

$F\left(d\right)$: represents the percentage of positioning errors that fall within

distance d, which is used to generate all CDF curves in Figure 5.

### 3.5. Loss Curve Analysis

**Figure 6:**
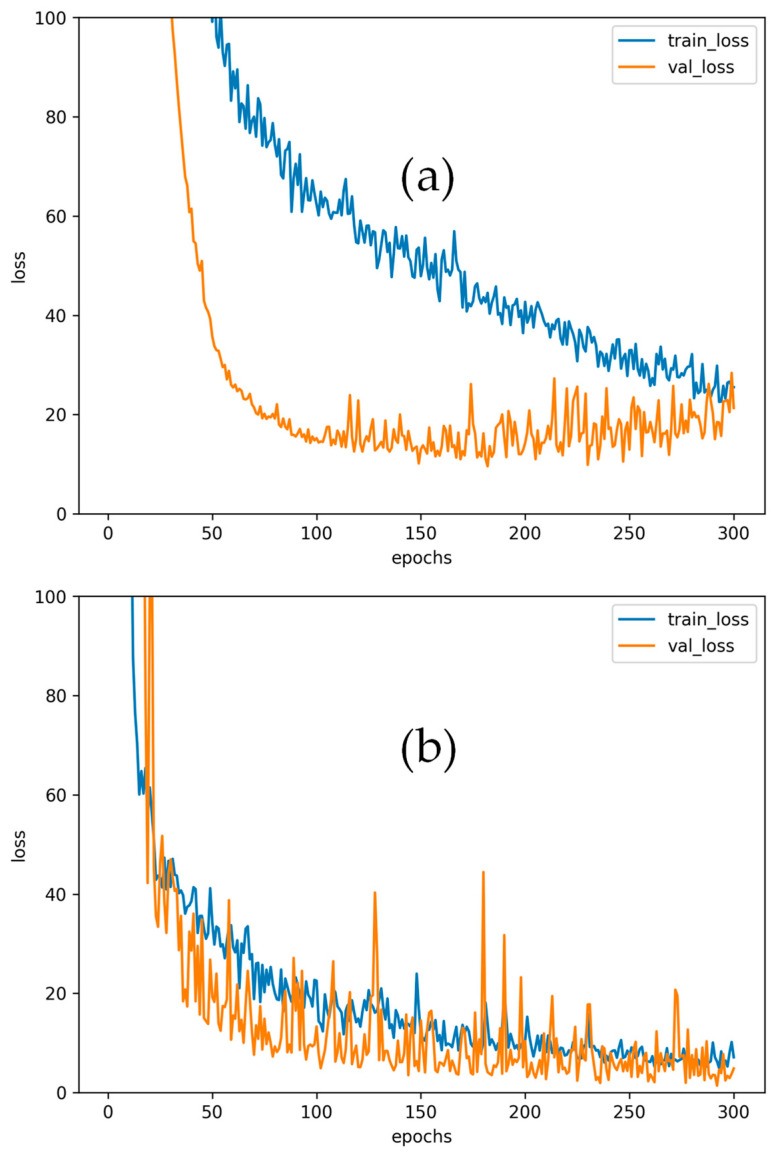
Training and validation loss curves of the (**a**) ANN and (**b**) CNN models at the 225 cm receiver plane.

To further assess the training stability and verify that no overfitting occurred, Figure 6 shows the training and validation loss curves of the ANN and CNN models at the 225 cm receiver plane. Both models exhibit smooth convergence behavior, where the training and validation losses decrease steadily and remain closely aligned throughout the training process. The CNN demonstrates slightly lower final loss values due to its superior ability to extract spatial dependencies from RSS inputs, while the ANN achieves faster initial convergence. The absence of divergence between the training and validation loss curves confirms that both models generalize well to unseen data. The applied dropout rate of 0.1 and sufficiently large dataset of 2240 samples per plane effectively prevent overfitting and ensure stable learning. Although a formal statistical test, such as a paired t-test, has not been performed, the observed performance improvements of the CNN + Pre-processing + PSO model consistently exceed one standard deviation of the corresponding baseline results. This indicates that the reduction in RMSE (approximately 15–20%) is not due to random variation but represents a practically significant improvement. Future work may include formal statistical hypothesis testing to further confirm the robustness and generalizability of these findings.

## 4. Conclusions

VLP is a promising indoor localization approach due to its high accuracy and cost-effectiveness. However, practical deployments suffer from signal attenuation, multipath reflections, and non-uniform illumination, making RSS-based VLP highly sensitive to model hyperparameters. In this work, we propose and demonstrate a VLP system that integrates RSS signal pre-processing, a CNN, and PSO for automated hyperparameter tuning. Here, a hybrid framework combining data pre-processing, CNN modeling, and PSO is developed to enhance positioning performance. Experimental results across three Rx planes (i.e., 200 cm, 225 cm, 250 cm) show that the CNN + pre-processing model improves positioning accuracy by 41.81%, 39.27%, and 18.76% at these three Rx planes, respectively. These percentage improvements are calculated using only the mean values in Table 2. With PSO-based hyperparameter tuning, the CNN model achieves further reductions in mean errors to 4.93 cm, 4.53 cm, and 3.87 cm, corresponding to additional accuracy gains of 13.81%, 17.03%, and 20.21% at these three Rx planes, respectively. These accuracy gains are calculated using only the mean values in Table 3. These findings demonstrate that integrating PSO-based hyperparameter tuning with CNN and RSS pre-processing significantly enhances positioning accuracy, reliability, and model robustness.

## Figures and Tables

**Table 1:** Candidate hyperparameters with constrained random initialization for PSO tuning.

Hyperparameter	Value
Dropout rate	{0.1, 0.4}
Learning rate	{1 × 10^−4^, 5 × 10^−3^}
Batch size	{16, 32, 64}
Optimizer	{Adam, SGD}

**Table 2:** The Improvement (%) columns quantify the relative reduction in mean positioning error after applying pre-processing, computed using Equation (6).

Planes (cm)	LR (cm)	LR + Pre. (cm)	LR Improvement (%)	ANN (cm)	ANN + Pre. (cm)	ANN Improvement (%)	CNN (cm)	CNN + Pre. (cm)	CNN Improvement (%)
200	11.33 ± 5.77	8.79 ± 5.59	22.42%	9.86 ± 7.29	6.36 ± 5.17	35.50%	9.83 ± 6.87	5.72 ± 4.24	41.81%
225	8.52 ± 4.47	8.10 ± 4.09	4.93%	7.36 ± 3.95	5.75 ± 4.20	21.88%	8.99 ± 7.36	5.46 ± 3.99	39.27%
250	6.03 ± 3.71	5.55 ± 2.90	7.96%	6.33 ± 3.64	5.37 ± 3.74	15.17%	5.97 ± 3.05	4.85 ± 3.07	18.76%

**Table 3:** Measured positioning errors (mean ± standard deviation) and relative accuracy gain (%) for (a) ANN models and (b) CNN models, using pre-processing with and without PSO.

**(a)**			
**Planes** **(cm)**	**ANN + Pre** **.** **(cm)**	**ANN + Pre + PSO** **(cm)**	**ANN** **Accuracy Gain** **(%)**
200	6.36 ± 5.17	6.05 ± 4.08	4.87
225	5.75 ± 4.20	4.62 ± 3.10	19.65
250	5.37± 3.74	4.09 ± 2.61	23.84
**(b)**			
**Planes** **(cm)**	**CNN + Pre** **.** **(cm)**	**CNN + Pre. + PSO** **(cm)**	**CNN** **Accuracy Gain** **(%)**
200	5.72 ± 4.24	4.93 ± 4.22	13.81
225	5.46 ± 3.99	4.53 ± 3.22	17.03
250	4.85 ± 3.07	3.87 ± 2.55	20.21

## Data Availability

The data presented in this study are available on request from the corresponding author.
